# The Role of Molecular Techniques on Malaria Control and Elimination Programs in Iran: A Review Article

**Published:** 2018

**Authors:** Saber GHOLIZADEH, Nazanin NASERI KARIMI, Sedigheh ZAKERI, Navid DINPARAST DJADID

**Affiliations:** 1. Cellular and Molecular Research Center, Urmia University of Medical Sciences, Urmia, Iran; 2. Medical Entomology Department, School of Public Health, Urmia University of Medical Sciences, Urmia, Iran; 3. Malaria and Vector Research Group (MVRG), Biotechnology Research Center (BRC), Pasteur Institute of Iran, Tehran, Iran

**Keywords:** Malaria, Molecular methods, Control, Elimination, Iran

## Abstract

**Background:**

The aim of this review was to describe the application of molecular methods in epidemiological aspects of malaria vectors, parasites, and human hosts in Iran and their critical role in malaria control and elimination programs.

**Methods:**

Medline, EMBASE, Web of Science, Scopus, and Google Scholar databases were searched systematically for original published papers on PCR, the molecular identification of malaria vectors, the molecular epidemiology of malaria, insecticide resistance, and drug-resistant parasites, in Iran. In total, 51 studies on molecular entomology and 36 studies on molecular parasitology of malaria and three on human host were selected.

**Results:**

Molecular methods are essential for improving the detection of malaria infection and monitoring antimalarial drugs and insecticide resistance in malaria elimination settings such as Iran.

**Conclusion:**

The application of molecular methods may be of particular interest for malaria control/elimination programs, for monitoring progress towards malaria elimination, and for optimal orientation of program activities.

## Introduction

Malaria is a vector-borne disease with an estimated 438000 deaths in 2015 (1). The disease is caused by *Plasmodium* parasites and transmitted exclusively through the bites of *Anopheles* species (1). Until recently, human malaria has been an anthroponotic disease caused by four *Plasmodium* species (2), and now *P. knowlesi* is the cause of zoonotic malaria, a type of malaria that naturally infects macaques in Southeast Asia as well as infects humans (3). Besides, additional species *P.* cynomolgi was identified by polymerase chain reaction (PCR) in a Malaysian patient and *P. simium*, a monkey malaria might be infecting humans in frontier regions of South America (4); however, whether this species can regularly infect humans is not known and further investigation is required (4–6).

Before 1951, the most prevalent *Plasmodium* species was *P. falciparum* (56.6%), followed by *P. vivax* (32.4%), and *P. malariae* (4.7%) in Iran (5, 6). However, malaria parasites presently circulating among malaria-endemic areas of Iran are *P. vivax* and *P. falciparum* (7) with the imported cases of *P. ovale* (8) and *P. malariae*. Regarding the prevalence of *Anopheles* species, 19 different species of *Anopheles* were presented in Iran (9). Among 30 Iranian *Anopheles* species (10), *An. maculipennis*, *An. sacharovi*, *An. stephensi*, *An. culicifacies*, *An. fluviatilis*, *An. dthali*, *An. superpictus*, and *An. pulcherrimus* account for the main malaria vectors (11–13).

Since the beginning of malaria control program in 1951, there have been limited tools for epidemiologists to study malaria. The primary controlling programs were including insecticide spraying, entomology survey, and environment management (7). Malariometric data and microscopic examination were two main case detection methods in Iranian malaria control program (11). During recent decades, PCR and rapid diagnostic test (RDT) have been applied for studying malaria in Iran (14–16), mainly due to quick and efficient analysis of large specimens, transport of DNA samples from field to lab and their subsequent storage, which is less prone to problems than collection and transfer of blood samples (17).

In the present investigation, we reviewed those published papers used new molecular techniques in order to assess the impact of molecular studies on challenges for malaria elimination in Iran.

## Methods

Eligible primary studies were those used at least one of the molecular techniques for the study of malaria parasites, vectors and/or human hosts in Iran. Published papers in non-English journals, conference abstracts, or unpublished data were not eligible for inclusion. PCR in any format using *Plasmodium*/*Anopheles*/human DNA or RNA amplification was included.

Electronic databases such as Medline, PubMed, EMBAS, Web of Science, Scopus, and Google Scholar were searched with the Key words: PCR, malaria, *Plasmodium*, *Anopheles*, insecticide resistance, drug resistance, vaccine candidate genes, and Iran until 2016.

A primary selection of studies was performed based on title and abstract using End-note X6 (Bld 6348), while ineligible and duplicate studies were removed. All researches considered conscious were included in the study and discrepancies were resolved by discussion or by advising through a panel of all authors.

## Results

The searches included 469 studies on “malaria in Iran”. When searched term was limited to “molecular malaria in Iran”, 105 studies were selected based on title and abstract. After removing ineligible and duplicate surveys, 90 studies were included in the current review. Investigations were categorized and reviewed in malaria parasites, malaria vector, and human host as follow:

### The molecular epidemiology of Plasmodium parasites

#### Correct identification of parasite species

More than one century after the discovery of malaria parasites, microscopic examination has been still the gold standard diagnostic method of malaria. However, in patients with low level of parasites and mixed infections, molecular techniques have reported the prevalence of infection around two folds more accurate than microscopic evaluation (18). For instance, for the first time, 18ssrRNA was used and nested PCR to detect *P. vivax* and *P. falciparum* in Southwestern Iran (19) and concluded that nested PCR can be a very useful complement to microscopic diagnosis in places where the transmission of these two parasites occurs (19). Two years later, the mixed infection of *P. falciparum* and *P. vivax* from north of the country was reported, it was free of malaria for almost twenty years (20). Eventually, the comparison between the results of microscopy and nested PCR detection of malaria parasites indicated that nested PCR has potential to detect a considerable higher number of suspected cases with mixed infections and is a suitable supplementary method to microscopy for accurate specific diagnosis of malaria species in field (20–22). However, malaria microscopy can be trusted as much as molecular techniques, if a skilled microscopist gets involved (23). On the other hand, the first imported *P. oval* malaria case in Iran was a 20-yr-old Nigerian soccer player in Bandar-Abbas, diagnosed by using molecular evidence (8). Recently, mitochondrial DNA (mtDNA) was used for detection of malaria parasites in saliva and urine of symptomatic patients. Saliva could be an alternative to blood in malaria diagnosis, in cases where repeat sampling is required (24).

#### Molecular detection of drug resistance

Since 2007, due to the prevalence of CQ-resistant parasites, the National Guidelines for Malaria Diagnosis and Treatment have changed from CQ to SP plus artesunate (AS) as the first-line and artemether-lumefantrine (Coartem®) as the second-line treatment in Iran (25). Therefore, to provide applied data to drug policy decision-makers for control and elimination of malaria, molecular techniques have been widely used for the detection of mutations associated with drug resistance since 2002 in Iran. Quinolone resistance in Iranian P*. falciparum* has been reported using *pfcrt*, *pfmdr1*, and *pfmrp* genes as molecular markers (26, 27). CQ, as the first-line drug, is inadequate for treatment of uncomplicated falciparum malaria and must be withdrawn from the current treatment strategy in Iran (27, 28). However, sulfadoxine/pyrimethamine (SP) remains efficacious for treatment of uncomplicated falciparum malaria when using *dhfr* and *dhps* markers (28–33). Molecular detection of single nucleotide polymorphisms (SNPs) in 22 blood samples from falciparum patients with CQ failure has demonstrated that alleles 184, 1034, 1042, and 1246 cannot serve as markers for CQ resistance in Iran (34). However, seven years after the adoption of SP-AS, as the first-line treatment in Iran, SP remained effective for treatment of uncomplicated falciparum malaria, as a partner drug with AS in Iran (25).

In 2008, ACT was adapted as the first-line anti-malarial therapy of *P. falciparum* in Iran. The detected SNPs were not significantly frequent in both unexposed and exposed examined isolates and required more consideration for the possible association of *pfatpase6* S769N gene with resistance among *P. falciparum* isolates (35). However, at that time, none of the potential mutations were associated with artemisinin, and its derivatives resistance was significantly changed (36).

The more availability of SP as the first-line treatment, *P. vivax* isolates were more exposed to SP, and the selection or the spread of resistant *pvdhfr* and *pvdhps* alleles might increase in the near future in this region (37). In addition, the genetic diversity of *dhfr* has been reported among *P. vivax* isolates collected from Hormozgan Province, Iran (38). Moreover, four years after introducing SP as the first-line antimalarial drug in Iran, the frequency of parasites carrying *pvdhfr*/*pvdhps* pure mutations increased from 0% in 2006 to 2.1% in 2010. SP could be effective in treatment against the erythrocytic stages of vivax malaria in Iran; however, the increased frequency of mutant haplotypes in Iran since 2006 has been worrying and indicates the emergence of drug-tolerant/resistant *P. vivax* isolates in Iran in near future (39). Therefore, to use optimally the existing antimalarial drugs and to overcome the resistance, it is necessary to identify the pattern of drug tolerance/resistance in all malaria settings of Iran.

#### Malaria vaccine candidates

There are currently no licensed vaccines with high efficacy, and the only licensed malaria vaccine, RTS, S, showed moderate efficacy (40, 41), however, Block5 of the *P. vivax* merozoite surface protein-1(PvMSP-1) is a marker of genetic polymorphism (42, 43). The polymorphism of PvMSP-1 block 5 was investigated among Iranian isolates and provided three distinct sequence types in the Iranian *P. vivax* population (14). Further, the existence of several *P. vivax* strains in Hormozgan Province of Iran based on SSCP-PCR in MSP-1 gene (44). Moreover, *msp-3α* as an adequate, applicable and easily was used target gene for molecular epidemiology studies of *P. vivax* isolates without the need for further sequencing analysis (45). Moreover, *pvmsp-*3α was reported as a useful method for determining the polymorphism of biotype A of *pvmsp-*3α gene, and *pvmsp*-3β gene cannot be a suitable marker for detection of *P. vivax* in blood sample (46, 47). The study on the relapse risk of vivax malaria in Hormozgan Province of Iran revealed the presence of reinfection or relapse in Iranian *P. vivax,* as well as the detection of *P. vivax* Chesson genotype, for the first time, in Iran (48)

Sequence analysis of the carboxyl (C)-terminal region of *pfmsp-1*, a potential malaria vaccine antigen, in Iranian *P. falciparum* clinical isolates has indicated limited antigenic diversity and thus supports the potential utility of the C-terminal region of *pfmsp*-1 in designing polyvalent vaccine constructs (49). The sequence diversity of the C-terminal region of *P. falciparum* MSP-1 was reported in Southern Iran (50), which was similar to the results obtained (51). In addition, allelic dimorphism in *P. falciparum* (Camp and FCR-3) based on erythrocyte-binding antigen-175 (EBA-175) gene was reported in the south-east of Iran (52).

Until 2006, there was no information on the genetic diversity of *P. vivax* using the CSP, a leading vaccine candidate, in *P. vivax* populations circulating in Iran. PCR-RFLP analysis on isolates collected in the temperate northern and in the tropical southern endemic areas of Iran revealed that the *P. vivax* parasites collected in the northern area were VK210 type, whereas the parasites collected in the southeastern regions were of both VK210 and VK247 types (53).

The region II of *P. vivax* Duffy binding protein (PvDBP-II) is a major target for development of naturally acquired immunity, and sequence polymorphisms in PvDBP-II may inhibit antibodies recognition (54, 55). Therefore, sequence analysis of PvDBP-II polymorphism among *P. vivax* populations in Iran showed genetic polymorphism (56, 57).

The apical membrane antigen-1 (AMA-1) is the latest studied malaria vaccine candidate gene in Iran. Analysis of intra-population diversity has revealed relatively high nucleotide and haplotype diversity at the *P. falciparum ama*-1 domain I of Iranian isolates (58). However, genetic analysis in *ama-1* among Iranian *P. vivax* isolates showed limited antigenic diversity. Most of the detected mutations were located outside B-cell epitopes (59, 60).

### The molecular ecology of Anopheline mosquitoes

#### Molecular identification of species and sibling species

Genetic analysis of rDNA-ITS2 and RAPD loci in field populations of Asian malaria vector, *An. stephensi*, revealed that this species could be considered as a single species with different biological and ecological forms in different zoogeographical zones of Iran (61). However, genetic structure analysis of *An. stephensi* biological forms in south and southeastern of Iran using mtDNA cytochrome oxidase subunits I and II showed that except for a few substitutions in COII, all three forms and populations were nearly identical (COI-COII)(62, 63). Recently, *An. stephensi* odorant-binding protein1 (AsteObp1) gene (intron I region) has been introduced as a new molecular marker for the molecular identification of mysorensis, intermediate, and type forms of the Asian main malaria vector (64).

*An. maculipennis* is the principle malaria vector in Europe and the Mediterranean. In 2002, the sequence of *An. persiensis* was identified from Rasht (Guilan Province) and Amol city in Mazandaran Province. Later, it was described (2003) as the first culicid, characterized and named principally on the basis of DNA evidence, from the northern Caspian Sea littoral provinces of Guilan and Mazandaran, Iran (65, 66). The most comprehensive molecular identification of *An. maculipennis* in Northern Iran revealed that among the six Iranian members of the maculipennis complex, *An. atroparvus*, *An. labranchiae*, *An. messeae*, *An. maculipennis*, *An. persiensis*, and *An. sacharovi*, the first three species are new records for Northern Iran (66).

There are limited studies on molecular identification of *A. culicifacies* in Iran, using rDNA-ITS2 as well as COI and COII, the presence of species A as well as A and B was reported in Iran (67, 68). rDNA-ITS2 sequence analysis on *An. fluviatilis* revealed the presence of Y sibling species in Iran (69), whereas *An. fluviatilis* V form was reported from Iran when using D3 (70). On the other hand, recent studies based on D3 sequence analysis have reported the presence of U and T sibling species of *An. fluviatilis* in Iran (71, 72). COI sequences analysis in south and southeastern parts of Iran confirmed the presence of these two species (73). However, ITS2 sequences analysis of Iranian Anophelines showed the presence of four different sequences of *An. fluviatilis* from Iran in the GenBank (10).

*An. superpictus* is the most widespread malaria vector in Iran. Morphological and molecular analyses of mtDNA COI-COII region in eight provinces of Iran have shown that two distinct morphological forms (A and B) and at least three genotypes (X, Y, and Z) of this species are distributed in Iran (74). However, a molecular survey in sympatric and allopatric populations of *An. superpictus*, using ITS2 sequences, has revealed 32.3% variation as well as a length polymorphism (357 vs. 378 bp) in the ITS2 region among the populations but not among morphological forms (75). The latest publication on Iranian anophelines has reported the presence of *An. superpictus* and *An. superpictus B* sequences in the GenBank, submitted from Iran with 69%–70% sequence similarity (10). Phylogenetic analysis based on neighbor-joining and maximum likelihood algorithms showed that this species is more closely related to *An. stephensi* and *An. pulcherrimus*; therefore, rather than being a cryptic species complex, *An. superpictus* and *An. superpictus B* are not sister species (10).

#### Molecular identification of Plasmodium parasites in Anopheles mosquitoes

Nested-PCR was applied, for the first time, for the detection and identification of malaria parasites in mosquitoes in Iran and reported 0.22% infection of *An. stephensi* to *P. vivax* (76). Later, the infection of *An. stephensi* and *An. culicifacies* mosquitoes (from Minab and Iranshahr, respectively) with *P. vivax* and the co-infection of *An. stephensi* with both *P. vivax* and *P. falciparum* were reported (77). Recently, the infection of *P. falciparum* was reported within *An. hyrcanus* collected from Fooman district in Guilan Province, North of Iran (78). Finally, the susceptibility of *An. stephensi* mysorensis to *P. vivax* VK210 haplotype, VK210B, was confirmed for the first time in Iran (79).

#### Molecular mechanisms of insecticide resistance

Sequence analysis of segment 6 of domain II of the para type voltage-gated sodium channel between pyrethroid-selected strain of *An. stephensi* from Dubai (DUB-R) and the standard susceptible strain showed leucine to phenylalanine amino acid substitution in the pyrethroid-resistant strain (80). Subsequently, this putative *kdr* mutation was studied in other Iranian malaria vectors, including *An. sacharovi*, *An. culicifacies*, *An. maculipennis*, and *An. hyrcanus* and *kdr*-related substitution were detected only in *An. sacharovi* and *An. Culicifacies* (78, 81, 82). Sequence comparison of the *GSTe2* (glutathione S-transeferase e2) coding regions among field-collected *An. stephensi*, *An. fluviatilis*, and *An. culicifacies* with *An. gambiae* revealed that despite nucleotide variation, none of which had led to any amino acid substitution, within these three main malaria species (83, 84).

#### Characterization of mosquito blood meals

For detection of blood meal DNA in killed *An. stephensi* and *Culex quinquefasiatus*, the preservation of samples in −20 °C could increase successful PCR production (85). However, the analysis of mosquito’s blood meals using cytochrome B sequences and *Xho*I restriction enzyme distinguished human blood from other vertebrates (86). Recently, determination of ABO group ratio in the residents as well as ABO group preference of malaria vectors in two malaria-endemic areas in south of Iran using mtDNA-cytB PCR-RFLP revealed the high prevalence of O group in this region. However, due to the low number of human blood-fed specimens, the ABO host choice of the mosquitoes remains unknown (87).

## Discussion

The combination of conventional and molecular techniques will be helpful to ascertain how malaria incidence is affected by parasites, vectors, and human host populations. Molecular studies on *Plasmodium* in Iran appear to be focused on the molecular detection and monitoring of parasites spp. and drug resistance, respectively. Therefore, accurate diagnosis, effective treatment, detection of mixed infections and asymptomatic cases, determination of the types of circulating parasites, and identification of imported species and strains from other endemic areas have led to prevent malaria epidemic and have facilitated the success of the malaria elimination program. The molecular monitoring of drug resistance in *P. falciparum* has been ongoing since 2002 in Iran, and the related data have led to *change* the national *malaria treatment* policy since 2005 in the country.

Most of the anophelines malaria vectors in Iran belong to complex species, including, *An. culicifacies*, *An. fluviatilis*, *An. maculipennis*, and *An. pulcherrimus*. Some other species such as *An. superpictus* and *An. stephensi* could be categorized as candidate complex species.

## Conclusion

Therefore, more molecular studies using different markers are needed regarding possible divergences among populations of these malaria vectors. The aim of insecticide resistance research using molecular techniques is improvement of our understanding of the insecticide resistance mechanisms at the genomic DNA level, the identification of mutations involved in the phenotype, and development of the tools to manage or overcome resistance. Despite limited studies on molecular resistance genes among Iranian malaria vectors, development of molecular diagnostics to facilitate the early detection and monitoring of insecticide resistance is one of the most important gaps in molecular insecticide resistance research, which is a need covered.
